# Supplementary material for: Modeling glioblastoma heterogeneity as a dynamic network of cell states
Source: Mol Syst Biol. 2021 Sep 16;17(9):e10105. doi: 10.15252/msb.202010105 (PMC8444284; doi:10.15252/msb.202010105)
Supplement: Supplementary file 6 — Source Data for Figure 5 [file MSB-17-e10105-s004.zip › Figure5A_sourcedata/GSEA_3071/hallmarks_stateI.GseaPreranked.1623742531165/HALLMARK_E2F_TARGETS.html]

Details for gene set HALLMARK\_E2F\_TARGETS[GSEA]

|  || Dataset | state13071 |
| Phenotype | NoPhenotypeAvailable |
| Upregulated in class | na\_neg |
| GeneSet | HALLMARK\_E2F\_TARGETS |
| Enrichment Score (ES) | -0.5344314 |
| Normalized Enrichment Score (NES) | -2.9280686 |
| Nominal p-value | 0.0 |
| FDR q-value | 0.0 |
| FWER p-Value | 0.0 |
Table: GSEA Results Summary

  

Fig 1: Enrichment plot: HALLMARK\_E2F\_TARGETS      
 Profile of the Running ES Score & Positions of GeneSet Members on the Rank Ordered List

  

| PROBE | GENE SYMBOL | GENE\_TITLE | RANK IN GENE LIST | RANK METRIC SCORE | RUNNING ES | CORE ENRICHMENT || 1 | PRDX4 |  |  | 494 | 0.519 | -0.1397 | No |
| 2 | MTHFD2 |  |  | 588 | 0.459 | -0.1613 | No |
| 3 | CDKN2A |  |  | 983 | 0.331 | -0.2739 | No |
| 4 | MSH2 |  |  | 1438 | -0.252 | -0.4052 | No |
| 5 | CTCF |  |  | 1450 | -0.253 | -0.4052 | No |
| 6 | RAD1 |  |  | 1475 | -0.255 | -0.4090 | No |
| 7 | CTPS1 |  |  | 1540 | -0.261 | -0.4246 | No |
| 8 | STAG1 |  |  | 1624 | -0.270 | -0.4457 | No |
| 9 | SMC3 |  |  | 1650 | -0.274 | -0.4496 | No |
| 10 | EED |  |  | 1694 | -0.279 | -0.4587 | No |
| 11 | RPA1 |  |  | 1717 | -0.282 | -0.4616 | No |
| 12 | KIF2C |  |  | 1735 | -0.284 | -0.4630 | No |
| 13 | CCNB2 |  |  | 1757 | -0.288 | -0.4655 | No |
| 14 | POLD2 |  |  | 1820 | -0.296 | -0.4800 | No |
| 15 | MLH1 |  |  | 1869 | -0.301 | -0.4904 | No |
| 16 | RAN |  |  | 1907 | -0.306 | -0.4974 | No |
| 17 | TIPIN |  |  | 1942 | -0.312 | -0.5034 | No |
| 18 | KPNA2 |  |  | 1944 | -0.312 | -0.4997 | No |
| 19 | CDC25A |  |  | 1953 | -0.313 | -0.4980 | No |
| 20 | DDX39A |  |  | 2043 | -0.324 | -0.5202 | No |
| 21 | LYAR |  |  | 2046 | -0.324 | -0.5166 | No |
| 22 | CNOT9 |  |  | 2047 | -0.325 | -0.5124 | No |
| 23 | SNRPB |  |  | 2064 | -0.328 | -0.5130 | No |
| 24 | CDKN1A |  |  | 2083 | -0.332 | -0.5140 | No |
| 25 | CCNE1 |  |  | 2087 | -0.332 | -0.5106 | No |
| 26 | CHEK1 |  |  | 2108 | -0.336 | -0.5122 | No |
| 27 | PLK4 |  |  | 2167 | -0.346 | -0.5249 | No |
| 28 | GSPT1 |  |  | 2177 | -0.348 | -0.5231 | No |
| 29 | MXD3 |  |  | 2201 | -0.352 | -0.5254 | No |
| 30 | PLK1 |  |  | 2206 | -0.353 | -0.5220 | No |
| 31 | ILF3 |  |  | 2222 | -0.358 | -0.5218 | No |
| 32 | SMC1A |  |  | 2240 | -0.361 | -0.5222 | No |
| 33 | POLE |  |  | 2252 | -0.364 | -0.5208 | No |
| 34 | PRPS1 |  |  | 2267 | -0.366 | -0.5202 | No |
| 35 | DONSON |  |  | 2316 | -0.375 | -0.5296 | Yes |
| 36 | CKS1B |  |  | 2332 | -0.380 | -0.5291 | Yes |
| 37 | NOP56 |  |  | 2333 | -0.380 | -0.5242 | Yes |
| 38 | BUB1B |  |  | 2335 | -0.381 | -0.5196 | Yes |
| 39 | MCM2 |  |  | 2349 | -0.383 | -0.5185 | Yes |
| 40 | PSIP1 |  |  | 2364 | -0.386 | -0.5177 | Yes |
| 41 | PRIM2 |  |  | 2409 | -0.394 | -0.5256 | Yes |
| 42 | WEE1 |  |  | 2426 | -0.397 | -0.5252 | Yes |
| 43 | POLA2 |  |  | 2452 | -0.401 | -0.5275 | Yes |
| 44 | CKS2 |  |  | 2466 | -0.404 | -0.5261 | Yes |
| 45 | AURKA |  |  | 2467 | -0.404 | -0.5209 | Yes |
| 46 | CSE1L |  |  | 2512 | -0.417 | -0.5286 | Yes |
| 47 | SPAG5 |  |  | 2519 | -0.418 | -0.5249 | Yes |
| 48 | RACGAP1 |  |  | 2522 | -0.419 | -0.5201 | Yes |
| 49 | CDC20 |  |  | 2540 | -0.425 | -0.5197 | Yes |
| 50 | PAICS |  |  | 2545 | -0.426 | -0.5154 | Yes |
| 51 | NCAPD2 |  |  | 2563 | -0.431 | -0.5148 | Yes |
| 52 | SSRP1 |  |  | 2580 | -0.434 | -0.5140 | Yes |
| 53 | CIT |  |  | 2604 | -0.440 | -0.5151 | Yes |
| 54 | UBR7 |  |  | 2611 | -0.442 | -0.5112 | Yes |
| 55 | DCK |  |  | 2636 | -0.449 | -0.5125 | Yes |
| 56 | EIF2S1 |  |  | 2638 | -0.450 | -0.5070 | Yes |
| 57 | DUT |  |  | 2640 | -0.450 | -0.5015 | Yes |
| 58 | DCTPP1 |  |  | 2644 | -0.451 | -0.4965 | Yes |
| 59 | EXOSC8 |  |  | 2659 | -0.456 | -0.4948 | Yes |
| 60 | NME1 |  |  | 2669 | -0.463 | -0.4915 | Yes |
| 61 | KIF22 |  |  | 2683 | -0.468 | -0.4893 | Yes |
| 62 | CDCA8 |  |  | 2695 | -0.470 | -0.4865 | Yes |
| 63 | MMS22L |  |  | 2698 | -0.471 | -0.4810 | Yes |
| 64 | MCM6 |  |  | 2699 | -0.471 | -0.4749 | Yes |
| 65 | MELK |  |  | 2709 | -0.474 | -0.4714 | Yes |
| 66 | TUBG1 |  |  | 2715 | -0.475 | -0.4668 | Yes |
| 67 | RPA3 |  |  | 2730 | -0.480 | -0.4647 | Yes |
| 68 | TIMELESS |  |  | 2752 | -0.487 | -0.4647 | Yes |
| 69 | UNG |  |  | 2758 | -0.489 | -0.4599 | Yes |
| 70 | BRCA2 |  |  | 2772 | -0.495 | -0.4573 | Yes |
| 71 | SRSF2 |  |  | 2782 | -0.497 | -0.4536 | Yes |
| 72 | SLBP |  |  | 2792 | -0.499 | -0.4498 | Yes |
| 73 | ASF1B |  |  | 2824 | -0.510 | -0.4524 | Yes |
| 74 | DSCC1 |  |  | 2828 | -0.512 | -0.4467 | Yes |
| 75 | CDCA3 |  |  | 2829 | -0.513 | -0.4400 | Yes |
| 76 | SPC24 |  |  | 2836 | -0.514 | -0.4352 | Yes |
| 77 | HMGB3 |  |  | 2839 | -0.516 | -0.4291 | Yes |
| 78 | TOP2A |  |  | 2842 | -0.517 | -0.4230 | Yes |
| 79 | E2F8 |  |  | 2847 | -0.519 | -0.4175 | Yes |
| 80 | LIG1 |  |  | 2848 | -0.520 | -0.4108 | Yes |
| 81 | TRIP13 |  |  | 2865 | -0.526 | -0.4088 | Yes |
| 82 | RAD21 |  |  | 2866 | -0.527 | -0.4020 | Yes |
| 83 | ANP32E |  |  | 2877 | -0.530 | -0.3981 | Yes |
| 84 | PTTG1 |  |  | 2895 | -0.540 | -0.3961 | Yes |
| 85 | CDKN2C |  |  | 2903 | -0.544 | -0.3912 | Yes |
| 86 | TRA2B |  |  | 2933 | -0.563 | -0.3925 | Yes |
| 87 | KIF4A |  |  | 2953 | -0.574 | -0.3907 | Yes |
| 88 | TACC3 |  |  | 2956 | -0.575 | -0.3839 | Yes |
| 89 | POP7 |  |  | 2980 | -0.588 | -0.3831 | Yes |
| 90 | TCF19 |  |  | 3019 | -0.613 | -0.3865 | Yes |
| 91 | GINS4 |  |  | 3021 | -0.615 | -0.3788 | Yes |
| 92 | DNMT1 |  |  | 3035 | -0.622 | -0.3747 | Yes |
| 93 | AURKB |  |  | 3056 | -0.639 | -0.3723 | Yes |
| 94 | JPT1 |  |  | 3062 | -0.644 | -0.3655 | Yes |
| 95 | CDKN1B |  |  | 3065 | -0.646 | -0.3578 | Yes |
| 96 | TMPO |  |  | 3088 | -0.666 | -0.3557 | Yes |
| 97 | MAD2L1 |  |  | 3099 | -0.675 | -0.3499 | Yes |
| 98 | NASP |  |  | 3106 | -0.682 | -0.3429 | Yes |
| 99 | RFC3 |  |  | 3108 | -0.683 | -0.3344 | Yes |
| 100 | PA2G4 |  |  | 3116 | -0.690 | -0.3275 | Yes |
| 101 | LMNB1 |  |  | 3121 | -0.692 | -0.3198 | Yes |
| 102 | POLD3 |  |  | 3122 | -0.695 | -0.3108 | Yes |
| 103 | SMC4 |  |  | 3126 | -0.699 | -0.3027 | Yes |
| 104 | MCM5 |  |  | 3132 | -0.706 | -0.2950 | Yes |
| 105 | GINS1 |  |  | 3137 | -0.713 | -0.2870 | Yes |
| 106 | CDK1 |  |  | 3149 | -0.727 | -0.2809 | Yes |
| 107 | HNRNPD |  |  | 3157 | -0.738 | -0.2735 | Yes |
| 108 | MKI67 |  |  | 3158 | -0.738 | -0.2639 | Yes |
| 109 | ORC6 |  |  | 3159 | -0.738 | -0.2544 | Yes |
| 110 | TK1 |  |  | 3162 | -0.745 | -0.2454 | Yes |
| 111 | STMN1 |  |  | 3171 | -0.751 | -0.2380 | Yes |
| 112 | MCM3 |  |  | 3182 | -0.761 | -0.2312 | Yes |
| 113 | USP1 |  |  | 3183 | -0.762 | -0.2213 | Yes |
| 114 | BRCA1 |  |  | 3185 | -0.765 | -0.2118 | Yes |
| 115 | PSMC3IP |  |  | 3194 | -0.772 | -0.2042 | Yes |
| 116 | BARD1 |  |  | 3201 | -0.783 | -0.1958 | Yes |
| 117 | BIRC5 |  |  | 3208 | -0.793 | -0.1874 | Yes |
| 118 | DIAPH3 |  |  | 3213 | -0.800 | -0.1782 | Yes |
| 119 | SPC25 |  |  | 3219 | -0.806 | -0.1693 | Yes |
| 120 | RFC2 |  |  | 3245 | -0.839 | -0.1659 | Yes |
| 121 | CCP110 |  |  | 3262 | -0.870 | -0.1594 | Yes |
| 122 | CENPM |  |  | 3269 | -0.881 | -0.1498 | Yes |
| 123 | HELLS |  |  | 3284 | -0.901 | -0.1423 | Yes |
| 124 | RANBP1 |  |  | 3290 | -0.911 | -0.1321 | Yes |
| 125 | CDKN3 |  |  | 3293 | -0.916 | -0.1208 | Yes |
| 126 | HMGA1 |  |  | 3299 | -0.931 | -0.1103 | Yes |
| 127 | PCNA |  |  | 3319 | -0.963 | -0.1035 | Yes |
| 128 | UBE2T |  |  | 3330 | -0.979 | -0.0938 | Yes |
| 129 | MYBL2 |  |  | 3340 | -1.006 | -0.0835 | Yes |
| 130 | MCM4 |  |  | 3341 | -1.006 | -0.0705 | Yes |
| 131 | EZH2 |  |  | 3342 | -1.015 | -0.0574 | Yes |
| 132 | DEK |  |  | 3344 | -1.025 | -0.0445 | Yes |
| 133 | RAD51AP1 |  |  | 3345 | -1.030 | -0.0312 | Yes |
| 134 | MCM7 |  |  | 3384 | -1.147 | -0.0276 | Yes |
| 135 | HMGB2 |  |  | 3390 | -1.184 | -0.0138 | Yes |
| 136 | UBE2S |  |  | 3396 | -1.205 | 0.0002 | Yes |
| 137 | ATAD2 |  |  | 3415 | -1.278 | 0.0114 | Yes |
| 138 | RRM2 |  |  | 3420 | -1.297 | 0.0270 | Yes |
Table: GSEA details [plain text format]

  

Fig 2: HALLMARK\_E2F\_TARGETS: Random ES distribution      
 Gene set null distribution of ES for **HALLMARK\_E2F\_TARGETS**
